# 

**Published:** 1840-12-12

**Authors:** 


### BIBLIOGRAPHICAL NOTICE.

*The American Medical Almanac for* 1841. By J. V. C. Smith, M. D., Editor of the Boston Medical and Surgical Journal.

This very useful little publication for the coming year has just been issued. It contains a vast amount of information—really multum in parvo—including, besides a mass of statistics, practical papers from Drs. Bowditch, Pennock, Fisher, Capen, and others.

